# ABX464: a good drug candidate instead of a magic bullet

**DOI:** 10.1186/s12977-015-0189-x

**Published:** 2015-07-28

**Authors:** Ben Berkhout, Yme U van der Velden

**Affiliations:** Laboratory of Experimental Virology, Department of Medical Microbiology, Center for Infection and Immunity Amsterdam (CINIMA), Academic Medical Center, University of Amsterdam, Meibergdreef 15, 1105 AZ Amsterdam, The Netherlands

## Abstract

Despite the significant number of antiviral drugs that are currently available in the clinics of developed countries, none of these affect the production stage of HIV-1 replication, more specifically the process of viral gene expression. For instance, several early attempts failed to generate inhibitors of the viral Tat protein, the small activator of viral transcription from the long terminal repeat (LTR) promoter. A recent study published in Retrovirology by Campos et al. presents a new small molecule inhibitor, ABX464, that targets the other small viral protein essential for viral gene expression, the Rev protein (Retrovirology 12:30, 2015). Targeting of multiple virus replication steps and silencing the generation of new progeny may be of particular value for current attempts to develop novel therapeutic strategies that provide a cure or functional cure for HIV-1 infection (Nat Rev Immunol 12: 607–614, 2012). We will briefly review some of the unique antiviral properties of ABX464, with the focus on its surprising ability to exhibit a sustained antiviral effect in a humanized mouse model. Although ABX464 may remain an important new addition to the anti-HIV arsenal, we do present a sobering alternative explanation for the long-lasting reduction in viral load after treatment cessation.

In the Retrovirology study [1], ABX464 was shown to block HIV-1 replication by preventing the export of unspliced viral RNA from the nucleus to the cytoplasm, a process that is regulated by the viral Rev protein. No direct ABX464-Rev interaction is proposed, but instead the drug seems to interact with an important cellular RNA regulatory complex. Although this indole drug class was originally described as targeting the splicing factor SRSF1 [2], ABX464 was found to bind directly to the cap binding complex (CBC). CBC controls RNA export and RNA splicing and interacts directly with the Rev protein [3]. In the new report, ABX464 was proposed to bind CBC and to specifically prevent Rev-mediated export of viral RNA, without interfering with cap binding or export of cellular transcripts [1]. ABX464 is indeed the first small molecule drug that (indirectly) targets the Rev function, although early gene therapy approaches already focused on Rev inhibition [4]. ABX464 is therefore the first member of a new drug class that blocks viral gene expression in cells that were already infected and in which the integrated provirus has already been established. The ability to block the production of new viruses seems an important asset for attempts to silence viral reservoirs.

The new report in Retrovirology adds several important new insights on ABX464’s mode of action. First and perhaps surprisingly given the proposed binding to the cellular CBC regulator, ABX464 does not seem to affect other RNA processes in human cells, thus avoiding an impact on the physiological state of the cell. Besides being non-toxic, ABX464 does not trigger the evolution of HIV-1 variants that are resistant to the drug action. A likely explanation is that the direct drug target is the cellular CBC and not a viral component, consistent with previous antiviral strategies [5]. It is therefore also likely that this drug will be active against various HIV-1 isolates and different subtypes, possibly even HIV-2 if it is similarly dependent on the CBC-Rev axis, but this was not tested. On top of this, a remarkable sustained antiviral effect was reported in a pre-clinical in vivo model system. Thus, ABX464 seems a drug with optimal properties: selective drug action, absence of drug-resistance, and a sustained antiviral effect that would be very welcome for cure attempts. However, we would like to present an alternative explanation for the sustained antiviral effect. To do so, we have to discuss in some detail the experimental conditions at which this surprising effect was observed.

Profound suppression of the HIV-1 YU2 strain was obtained by state of the art combined antiretroviral therapy (cART) in the humanized immune system (HIS) mouse model, but an immediate rebound of the viral load was observed after the treatment was stopped. ABX464 monotherapy also led to a significant reduction of the viral load, but virus suppression was slower and less complete than with cART. The perplexing result was that this antiviral effect lasted for several weeks after cessation of ABX464 treatment [1]. No such an effect has ever been described for other treatments. The explanation provided is that ABX464 prevents virus replication in macrophages, thereby blocking the establishment of a viral reservoir that will cause the rebound after stopping therapy.

There are several reasons why this interpretation is not likely. First of all, it seems quite unlikely that macrophages form the major cellular component of the HIV-1 reservoir [6, 7]. Second, ABX464 could never have blocked all reservoir formation as the drug was added only at day 52 after the experimental infection of the mice. In fact, all reservoirs will likely have been established already by that time. For instance, a recent macaque study indicated that reservoirs are seeded after just 3 days [8]. Although we certainly value the power of the HIS mouse model [9], this unique in vivo model remains rather variable and complex as each HIS mouse is generated individually and critical parameters such as engraftment and source of hematopoietic stem cells differ between experiments. We like to offer an alternative explanation for these striking results, also because such a sustained inhibitory effect after drug removal has never been described for antivirals, including ABX464, in more simple in vitro settings. Because ABX464 does only sub-optimally inhibit YU2 replication, it could be that most target cells have been depleted (by virus replication and/or immune reactions) after 100 days, the moment at which the therapy was stopped. The absence of sufficient target cells would provide a very simple explanation for the observed lack of viral rebound. Although the CCR5-tropic YU2 virus has been reported to sustain viral replication for 150–200 days in HIS mice without a noticeable decline in viral load [10, 11], another study [12] demonstrated an almost log10 reduction in viral load after 100 days of infection, which is in the range of the observed sustained reduction in viral load upon ABX464 withdrawal and which could be explained by a shortage of target cells. In fact, some of the additional data in the ABX464 study do support this alternative scenario. Close inspection of the results obtained for individual mice indicates that there is a correlation between the viral loads at cessation of therapy and the viral loads at the end of the experiment. This correlation may be imposed by the number of target cells available. One could obviously test this alternative explanation in many ways. The authors could for instance have refuted this alternative scenario by including an untreated YU2-infected group to monitor the CD4^+^ T cell dynamics throughout the experiment and at sacrifice. We believe that this commentary is relevant as the original study did not entertain any alternative explanations for the sustained drug action and the ABX464 antiviral was evaluated for the pharmacokinetic properties and biological safety in healthy volunteers by the ABIVAX biotech company in a Phase 1 study. No serious adverse events were scored and no clinically significant abnormal result was reported in physical examinations, laboratory tests, vital signs and ECG. A Phase 2 study of ABX464 in patients with HIV was recently initiated. Although we would immediately agree that ABX464 remains an interesting drug candidate that could form a welcome addition to the current classes of antivirals, some caution is warranted because the sustained drug action may be an artifact of the pre-clinical model system. The search continues for anti-latency drugs, but one should entertain and exclude indirect effects when a magical sustained drug action is observed.
